# Structural and functional characterization of gut microbiota in dyslipidemic patients from high-altitude Tibetan pastoral areas

**DOI:** 10.3389/fnut.2025.1676238

**Published:** 2025-10-08

**Authors:** Juanjuan Chen, Yuanhao Luo, Yun Hao, Qian Wang, Qi Wang

**Affiliations:** ^1^Cuiying Biomedical Research Center, The Second Hospital and Clinical Medical School, Lanzhou University, Lanzhou, China; ^2^The Second School of Clinical Medicine, Lanzhou University, Lanzhou, China

**Keywords:** dyslipidemia, Tibetan pastoral area, shotgun metagenomic sequencing, gut microbiota, microbial metabolic pathway prediction, metagenome-wide association study

## Abstract

**Introduction:**

Approximately 49.3% of individuals living at high altitudes suffer from dyslipidemia. Emerging evidence indicates that gut microbiota can regulate lipid metabolism and cholesterol homeostasis, but the composition and function of gut microbiota in dyslipidemic patients from Tibetan pastoral regions remain unclear.

**Methods:**

To address this, we enrolled a cohort consisting of 22 dyslipidemic patients and 33 healthy controls (HCs) from the Gannan Tibetan pastoral region (average altitude: 3,600 m). Phenotypic data, blood, and fecal samples were collected from all the participants for a metagenome-wide association study based on shotgun metagenomic sequencing.

**Results:**

Compared with HCs, dyslipidemic patients showed a significant reduction in gut microbial diversity. Specifically, the abundance of beneficial species—including *Faecalibacterium prausnitzii*, *Bifidobacterium adolescentis*, *Bifidobacterium longum*, *Bifidobacterium bifidum*, and *Parabacteroides distasonis*—was significantly decreased, while opportunistic pathogens such as *Veillonella parvula*, *V. tobetsuensis*, *Streptococcus oralis*, and *Streptococcus mitis* were notably enriched. Functional prediction revealed that pathways involved in glycolysis, starch degradation, and biosynthesis of L-methionine, L-arginine, L-lysine, L-citrulline, and L-threonine were significantly downregulated in dyslipidemic patients, whereas pathways for the biosynthesis of lipopolysaccharides, fatty acids, polyamines, and (Kdo)₂-lipid A were enriched. Correlation analysis showed that the enriched taxa in dyslipidemic patients were significantly positively associated with total cholesterol (TC), total triglycerides (TG), and low-density lipoprotein cholesterol (LDL-C). In contrast, the enriched functional pathways were significantly negatively correlated with TC but positively correlated with high-density lipoprotein cholesterol (HDL-C).

**Discussion:**

These findings clarify the alterations in gut microbiota composition and function in plateau-dwelling dyslipidemic populations and their associations with blood lipid levels, suggesting potential microbial biomarkers for hyperlipidemia in plateau environments.

## Introduction

Dyslipidemia, a common metabolic disease characterized by abnormal elevations in blood lipids such as cholesterol and triglycerides, is a critical risk factor for cardiovascular diseases (1). According to the World Health Organization (WHO), approximately 400 million people worldwide suffer from hyperlipidemia, while the overall prevalence rate of dyslipidemia among Chinese adults has reached 35.6% and has continued to rise (2). This trend poses a severe threat to public health, emerging as a major global challenge.

The gut microbiota participates in key physiological processes such as metabolism, immunity, and nutrient absorption, with close ties to the onset and progression of various diseases. Recent evidence highlights its pivotal role in regulating circulating lipids and lipid metabolism (3), where structural and functional alterations correlate strongly with hyperlipidemia development (4, 5). For instance, an altered intestinal environment may inhibit beneficial bacteria (e.g., *Bifidobacterium*, *Lactobacillus*, and butyrate-producing species) while promoting *Enterobacteria* proliferation, triggering dyslipidemia that further exacerbates microbial dysbiosis (4). Mechanistically, the gut microbiota influences blood lipid levels by modulating bile acid metabolism and short-chain fatty acid production, thereby regulating lipid absorption and metabolism (6). Additionally, high-altitude environments can induce gastrointestinal disorders and alterations in gut microbiota composition (7). A study on Tibetan children at different altitudes found that higher-altitude residents with lower body weights exhibit reduced microbial diversity, with *Prevotella* potentially suppressing obesity in high-altitude settings (8).

The Gannan Tibetan Autonomous Prefecture, located in southern Gansu Province, is a major Tibetan settlement where residents endure hypoxic, cold, low-humidity, and high-solar-radiation conditions, alongside a unique diet rich in protein and fat but low in carbohydrates. This dietary pattern, coupled with relatively limited local medical resources, elevates the risk of plateau-related cardiovascular diseases among Gannan Tibetans (9), including dyslipidemia, pulmonary morbidities (10), and memory dysfunction (11, 12), which are leading causes of death in the region.

Notably, specific environments shape distinct gut microbiota, facilitating adaptation to local conditions. For example, a study conducted in Lhasa (3,660 m) revealed that Tibetan men have a higher prevalence of hypertriglyceridemia, while women exhibit lower HDL-C, and all genders face elevated hypercholesterolemia rates (13). Similarly, among 1,415 residents at 1,500–2,000 m on the Yunnan-Kweichow Plateau, 49.3% had hyperlipidemia, with prevalences of 23.3% (hypercholesterolemia), 34.1% (hypertriglyceridemia), 17.5% (low HDL-C), and 9.0% (high LDL-C) (14). Conversely, higher-educated individuals in high-altitude areas show lower hypercholesterolemia and hyperglycemia rates compared to low-altitude counterparts (15). Additionally, Tibetans residing above 3,000 m for generations exhibit significantly lower plasma and intestinal lipopolysaccharide (LPS) levels than low-altitude Han populations (Tibetans: 2.65 ± 0.68 pg/mL vs. Han: 39.43 ± 7.13 pg/mL), even among coronary heart disease patients (5.70 ± 0.69 pg/mL vs. 52.14 ± 9.22 pg/mL) (2). This finding suggests that Tibetans may possess enhanced intestinal barrier function and a unique microbiota structure.

In summary, the Gannan Tibetan population—with its special dietary structure, living environment, and high incidence of hyperlipidemia and cardiovascular diseases—offers a unique model to study the gut microbiota-dyslipidemia relationship. An in-depth exploration of the structure and function of gut microbiota in Gannan Tibetan dyslipidemic patients could reveal the pathogenesis of hyperlipidemia, provide novel strategies for local prevention and treatment of hyperlipidemia, and hold significant scientific and clinical value.

## Methods and materials

### Enrollment of participants

**Figure 1:**
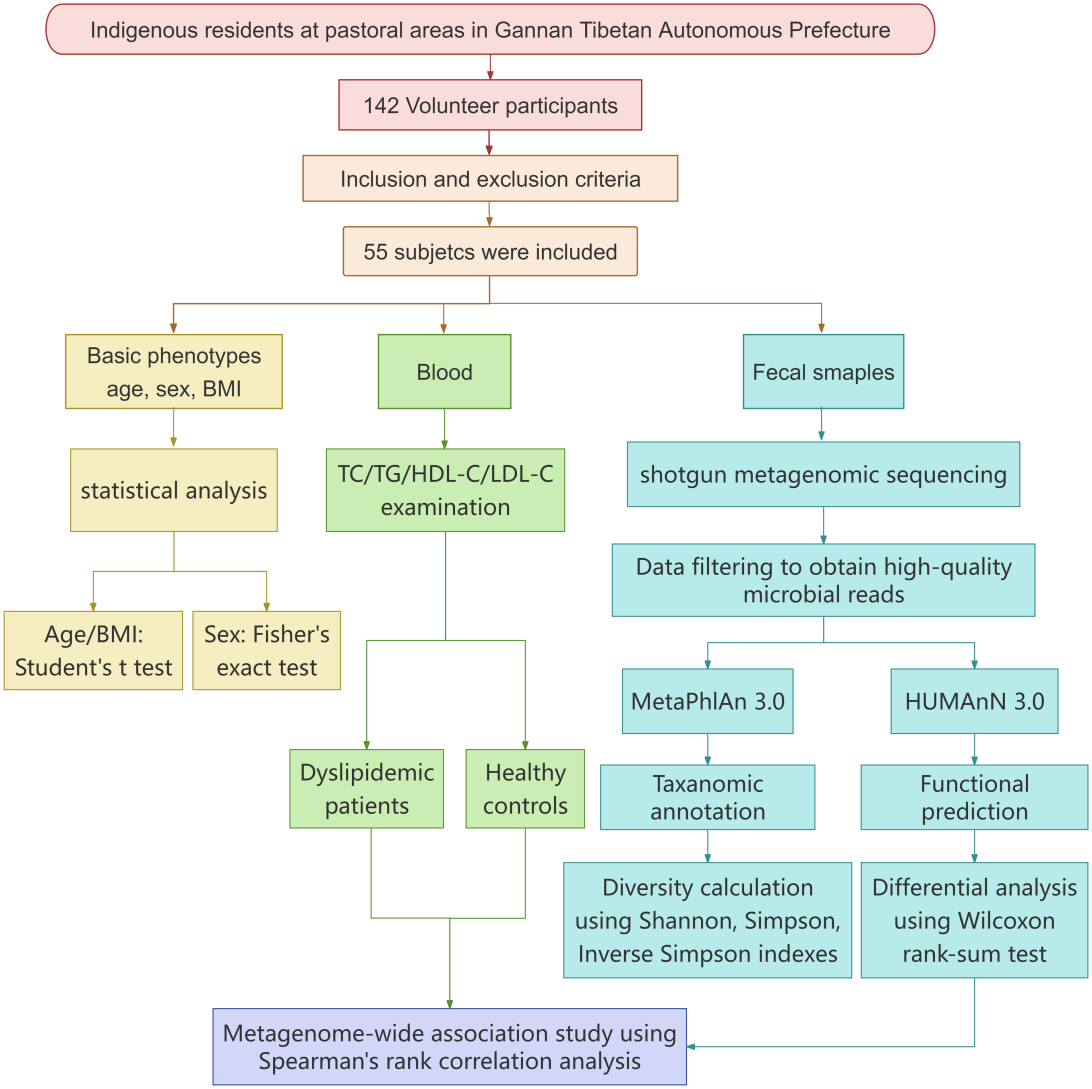
Flowchart of the experiment.

This study was approved by the Medical Ethics Committee of Lanzhou University Second Hospital (Approval No. 2021A-187) on 26 April 2021. All participants voluntarily signed the informed consent form. The entire research was conducted in strict accordance with the following flowchart, which covers key steps from experimental design to result validation (see Figure 1).

The inclusion and exclusion criteria are shown below:

#### Inclusion criteria

1. Local Tibetan residents from pastoral areas in Luqu County (altitude >3,500 m), Gannan Tibetan Autonomous Prefecture, Gansu Province.
2. Residents aged 18–65 years.
3. Patients diagnosed with dyslipidemia in accordance with the authoritative diagnostic criteria specified in the *Chinese Guidelines for the Prevention and Treatment of Dyslipidemia in Adults* (2023 Edition), i.e., meeting at least one of the following abnormal indicators: total cholesterol (TC) ≥5.2 mmol/L, triglyceride (TG) ≥1.7 mmol/L, high-density lipoprotein cholesterol (HDL-C) <1.0 mmol/L, or low-density lipoprotein cholesterol (LDL-C) ≥3.4 mmol/L.
4. Patients having maintained a relatively stable lifestyle (e.g., dietary structure, exercise habits) and an unchanged living environment for at least 3 months prior to the study.

#### Exclusion criteria

1. Patients having used antibiotics, probiotics, prebiotics, laxatives, weight-loss drugs, or other medications, including immunosuppressants (e.g., cyclosporine), lipid-lowering agents (e.g., statins, fibrates, niacin, or the traditional Chinese lipid-lowering preparation Xuezhikang), glucocorticoids, diuretics, or antipsychotic drugs (16) that may affect gut microbiota structure/function or lipid metabolism within the past month.
2. Those suffering from acute infectious diseases (e.g., acute respiratory tract infections, acute gastroenteritis), or being in the acute attack phase of chronic diseases (e.g., acute exacerbation of chronic obstructive pulmonary disease, diabetic ketoacidosis).
3. Those having severe organic diseases such as severe hepatic or renal insufficiency, cardiopulmonary dysfunction, or malignant tumors.
4. Those diagnosed with autoimmune diseases (e.g., systemic lupus erythematosus, rheumatoid arthritis) or immunodeficiency diseases.
5. Those having a history of gastrointestinal surgery (e.g., subtotal gastrectomy, intestinal resection) or suffering from chronic gastrointestinal diseases (e.g., inflammatory bowel disease, irritable bowel syndrome, peptic ulcer).
6. Those being pregnant or lactating.
7. Those having mental disorders or cognitive impairment, resulting in the inability to cooperate with research-related questionnaires, sample collection, or follow-up.
8. Those having allergic reactions to sample collection equipment, testing reagents, or other materials used in the study, which may affect research implementation or cause additional adverse reactions.
9. Those with incomplete collection of fecal and blood samples, or phenotypic data.

Using the aforementioned strict inclusion and exclusion criteria, a total of 142 local residents from pastoral areas in Luqu were recruited for the project. However, after rigorous screening based on these criteria, only 55 subjects were finally included in the study, with both fecal and blood samples successfully collected. Among them, 22 were diagnosed with dyslipidemia (HLP) and 33 were healthy controls (HCs).

### Phenotype characterization and sample collection

Demographic data, including age, sex, height, and weight, were recorded for all participants (Supplementary Table S1A). Venous blood samples were collected for biochemical analysis, encompassing measurements of HDL-C, LDL-C, TC, and TG levels.

For fecal sample collection and processing, approximately 5–10 g of stool samples were collected into sterile 1.8 mL tubes containing DNA stabilizer, immediately snap-frozen in liquid nitrogen, and stored at −80 °C until processing. After completion of sample collection, all fecal samples were transported to the laboratory on dry ice for metagenome extraction and metagenomic sequencing.

### Shotgun metagenomic sequencing and analysis

Shotgun metagenomic sequencing was performed following a previously described protocol (17), with detailed procedures described as follows:

#### Total DNA extraction and library construction

Total genomic DNA was extracted from each sample using the cetyltrimethylammonium bromide (CTAB) method—a standard protocol for isolating high-quality DNA from complex microbial communities in fecal samples.

The purity and concentration of the extracted total DNA were determined using a NanoDrop Spectrophotometer ND-1000 (Thermo Fisher Scientific Inc., Waltham, MA, United States). DNA purity was evaluated based on the A260/A280 ratio (1.8–2.0) and the A260/A230 ratio (2.0–2.2).

Metagenomic libraries were constructed using the TruSeq DNA PCR-Free Library Preparation Kit (Illumina, San Diego, CA, United States), a PCR-free workflow that reduced bias in microbial community representation. The quantity of each library was evaluated using a Qubit 2.0 fluorimeter (Invitrogen, Carlsbad, CA, United States) to ensure library concentrations met the minimum requirement (≥10 ng/μL) for subsequent sequencing.

#### Metagenomic sequencing and data filtering

Qualified libraries were subjected to paired-end sequencing on a BGI-SEQ500 platform with a read length of 2 × 150 bp. An average of ≥7.75 Gbp raw data was obtained per sample (Supplementary Table S1B).

Raw reads containing ≥50% low-quality bases (quality score ≤20) or >5 ambiguous bases were removed using FASTP. The remaining high-quality sequencing reads were aligned to the human genome (hg19) using bowtie2 with parameters (-m 100 -× 600 -v 7 -p 6 -l 30 -r 1 -M 4 -c 0.95), and human-matching reads were discarded. High-quality microbial reads were defined as clean reads and used for downstream analysis.

#### Taxonomic and functional annotation

MetaPhlAn 3.0 (MetaPhlAn, version 3.0, -input_type fastq - ignore_viruses - nproc 6) was used to generate the taxonomic profiling, including phylum-, genus-, and species-level profiles from high-quality clean reads (18) (Supplementary Table S1C). HUMAnN 3.0 (HUMAnN, version 3.0, -i input_clean_data -o output -threads 10 -memoryuse maximum -remove-temp-output) based on Uniref50 database was used to predict microbiota metabolic pathways (18) (Supplementary Table S1H).

#### Diversity analysis

Alpha diversity was calculated using Shannon, Simpson, and Inverse Simpson indices in R 4.0.3 [vegan package: diversity (data, index = “richness/Shannon/Simpson/InSimpson”)].

Beta diversity was assessed using Bray–Curtis dissimilarity (vegan:vegdist) and visualized via principal coordinates analysis (PCoA) (ape:pcoa). Permutational multivariate analysis of variance [PERMANOVA; vegan:adonis (dist~phe, permutations = 1,000)] was performed to test the significance of group differences in microbial composition.

The PERMANOVA [code: R 4.0.3: adonis (dist~phe, permutations = 1,000)] was performed based on the gut microbial species/genus abundance profile to study the differences between the two groups.

### Differential analysis

The Wilcoxon rank-sum test was applied to compare gut microbial taxa and predicted functional pathways between dyslipidemic patients and control groups. Student’s *t*-test was used to compare continuous clinical parameters (TC, TG, HDL-C, LDL-C, and age) between two groups, while Fisher’s exact test was used for the categorical variable sex.

### Metagenome-wide association study

Spearman’s rank correlation analysis was used to identify associations between the significantly modified gut microbial species/functional pathways and blood lipids.

## Results

### Significant differences in gut microbial composition between the two groups

**Figure 2:**
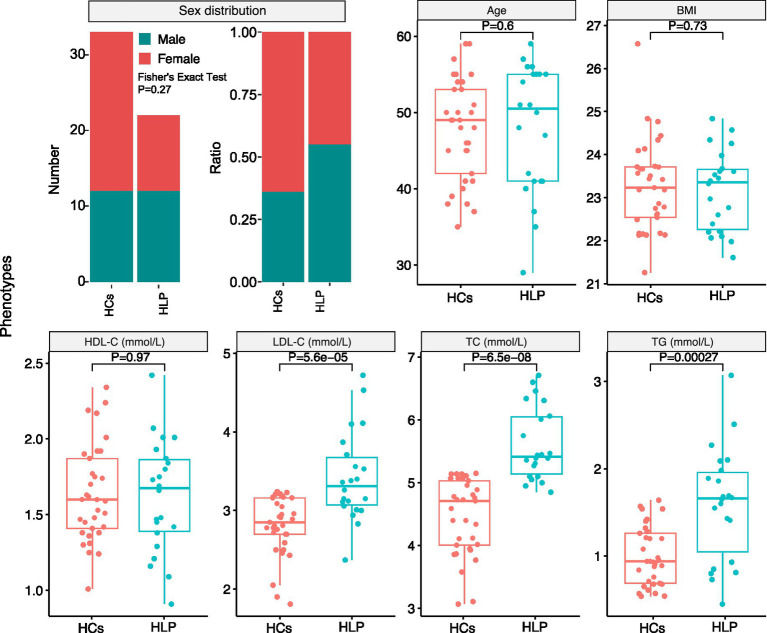
Statistical analysis of phenotypes between the hyperlipidemic patients (HLP) and healthy controls (HCs): sex distribution, age, body mass index (BMI), high-density lipoprotein cholesterol (HDL-C), low-density lipoprotein cholesterol (LDL-C), total cholesterol (TC), and triglycerides (TG).

A total of 22 dyslipidemic patients and 33 healthy controls (HCs) were enrolled, consisting of 31 women and 24 men (dyslipidemic group, F:M = 10:12; HCs group, F:M = 21:12). The overall mean body mass index (BMI) of the entire cohort was 23.22 ± 0.99 kg/m^2^ (dyslipidemic group: 23.16 ± 0.93 kg/m^2^; HCs group: 23.27 ± 1.04 kg/m^2^), and the overall mean age was 47.84 ± 7.33 years (dyslipidemic group: 47.95 ± 8.40 years; HCs group: 47.76 ± 6.66 years). A comparative analysis between the two cohorts revealed a significant increase in total cholesterol (TC), triglyceride (TG), and low-density lipoprotein cholesterol (LDL-C) levels in dyslipidemia, while no statistically significant variations were observed in high-density lipoprotein cholesterol (HDL-C), age, body mass index (BMI), or sex distribution (Figure 2).

**Figure 3:**
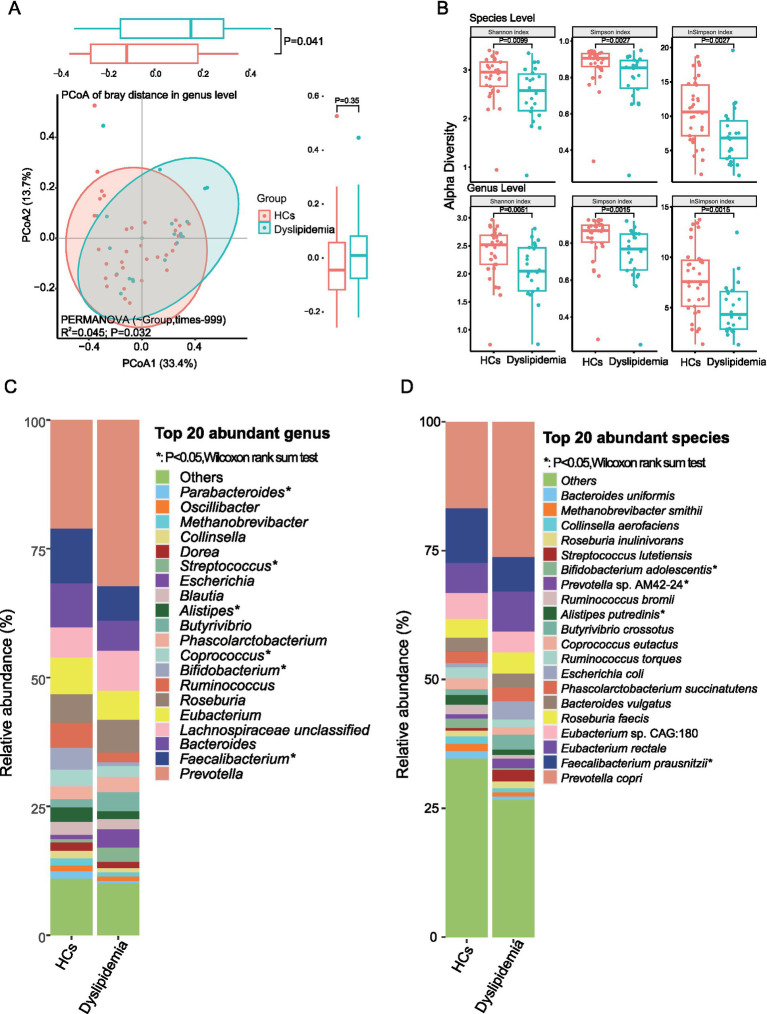
The gut microbiota of dyslipidemic individuals in plateau areas is found to be significantly different from that of healthy controls (HCs). **(A)** The PCoA reveals significant dissimilarities in the composition of gut microbiota between dyslipidemic patients and HCs. **(B)** The alpha diversity exhibits a significant decrease in dyslipidemic patients compared to HCs. **(C)** Differential analysis of the top 20 most abundant genera between two groups. **(D)** Differential analysis of the top 20 most abundant species between two groups.

Principal coordinate analysis (PCoA) based on Bray–Curtis distances of genus- and species-level profiles demonstrated significant differences in gut microbiota composition between dyslipidemic patients and HCs in plateau environments (Figure 3A, genus level: *R*^2^ = 0.042, *p* = 0.032; Supplementary Figure S1A, species level: *R*^2^ = 0.034, *p* = 0.041). Alpha diversity, as measured by the Shannon, Simpson, and Inverse Simpson indices, was significantly reduced in the gut microbiota of the dyslipidemic group compared to HCs at both the species and genus levels (Figure 3B, *p* < 0.01; Supplementary Table S1G).

To further characterize the specific differences in the gut microbial composition between the two groups, we analyzed the most abundant taxa. At the phylum level, Firmicutes, Bacteroidetes, Actinobacteria, Proteobacteria, and Euryarchaeota were the top five phyla, with mean relative abundance (RA) of 54.74, 37.30, 4.20, 2.36, and 1.16%, respectively (Supplementary Figure S1B and Supplementary Table S1D).

Among the top 20 most abundant genera—which collectively accounted for ~89% of the total genera in the sample set, including *Prevotella*, *Faecalibacterium*, *Bacteroides*, *Eubacterium*, *Roseburia*, *Ruminococcus*, and *Bifidobacterium*—the dyslipidemic group exhibited significantly decreased RA of *Faecalibacterium*, *Bifidobacterium*, and *Alistipes*, alongside a notable increase in *Phascolarctobacterium* (Figure 3C and Supplementary Table S1E).

At the species level, the top 20 most abundant species (collectively accounting for 68% of total RA) included *Prevotella copri*, *Faecalibacterium prausnitzii*, *Eubacterium rectale*, *Roseburia faecis*, *Bacteroides vulgatus*, *Phascolarctobacterium succinatutens*, *Alistipes putredinis*, and *Bifidobacterium adolescentis*. Among these, *F. prausnitzii*, *A. putredinis*, and *B. adolescentis* showed significantly reduced RA in the dyslipidemic group, whereas *P. succinatutens* and Prevotella sp. AM42-24 were significantly increased (Figure 3D and Supplementary Table S1F).

### Differential analysis of gut microbiota and their correlations with blood lipids

**Figure 4:**
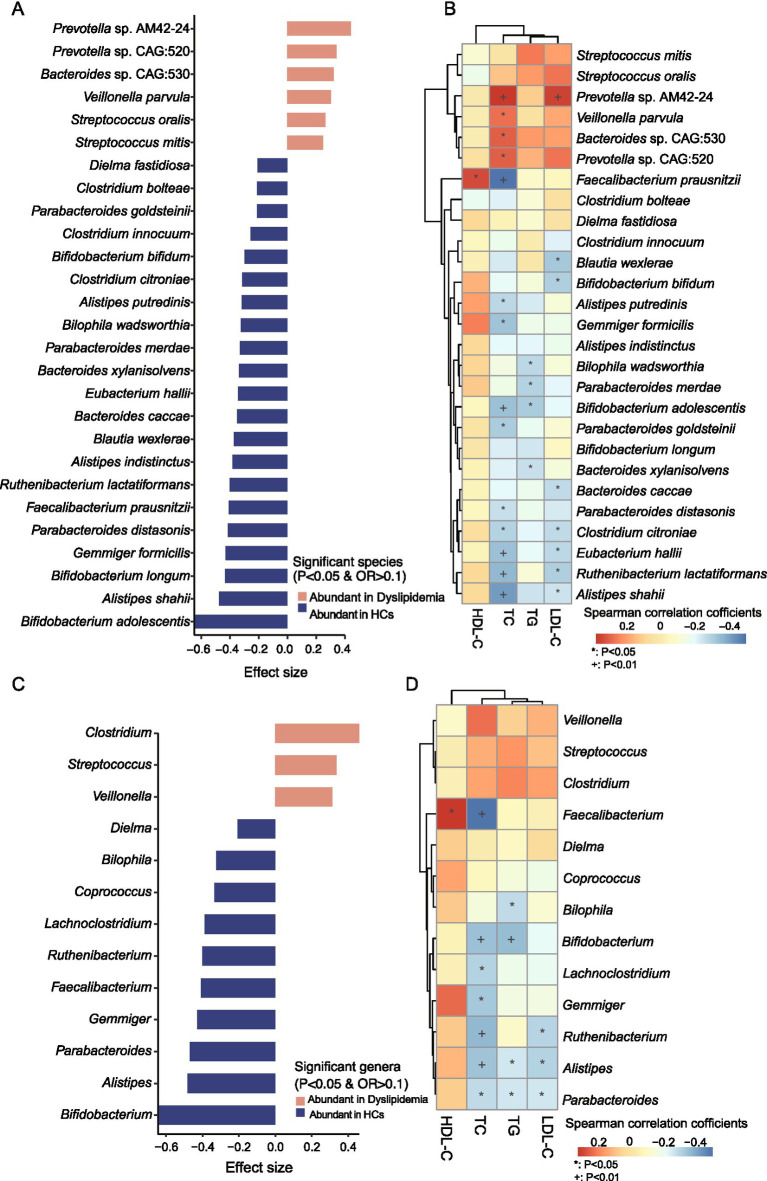
Identification of significantly distinct species and genera between two groups and their associations with blood lipid levels. **(A)** Significantly different species between the two groups. **(B)** Association study between the significantly different species and blood lipid levels. **(C)** Significantly different genera between two groups. **(D)** Association study between the significantly different species and blood lipid levels.

To further characterize the differences in gut microbiota composition between the two groups, we analyzed the taxa that exhibited significant variations. At the species level, 27 taxa showed marked differences: 21 species were significantly depleted in the dyslipidemic group compared to HCs, including *Bifidobacterium adolescentis*, *Alistipes shahii*, *Bifidobacterium longum*, *Gemmiger formicilis*, *Parabacteroides distasonis*, *Faecalibacterium prausnitzii*, *Ruthenibacterium lactatiformans*, *Alistipes indistinctus*, *Blautia wexlerae*, *Bacteroides caccae*, *Eubacterium hallii*, *Bacteroides xylanisolvens*, *Parabacteroides merdae*, *Bilophila wadsworthia*, *Alistipes putredinis*, *Clostridium citroniae*, *Bifidobacterium bifidum*, *Clostridium innocuum*, *Parabacteroides goldsteinii*, *Clostridium bolteae*, and *Dielma fastidiosa*; in contrast, six species were significantly enriched in dyslipidemia, namely *Streptococcus mitis*, *Streptococcus oralis*, *Veillonella parvula*, *Bacteroides* sp. CAG:530, *Prevotella* sp. CAG:520, and *Prevotella* sp. AM42-24 (Figure 4A).

At the genus level, 13 genera displayed significant differences: 10 genera were depleted in dyslipidemia relative to HCs, including *Bifidobacterium*, *Alistipes*, *Parabacteroides*, *Gemmiger*, *Faecalibacterium*, *Ruthenibacterium*, *Lachnoclostridium*, *Coprococcus*, *Bilophila*, and *Dielma*; whereas three genera (*Veillonella*, *Streptococcus*, and *Clostridium*) were significantly enriched (Figure 4C). At the phylum level, the relative abundance of Actinobacteria was significantly reduced in the dyslipidemic group (Supplementary Figure S1B).

Spearman’s rank correlation analysis was performed to explore associations between gut microbial composition, predicted functional pathways, and serum lipid levels (TC, TG, HDL-C, and LDL-C; Supplementary Table S1J). Several genera and species, which decreased in dyslipidemic patients, including *Bifidobacterium*, *Alistipes*, *Bilophila*, *Lachnoclostridium*, *Gemmiger*, *Ruthenibacterium*, *Parabacteroides*, *C. citroniae*, *E. hallii*, *R. lactatiformans*, *A. shahii*, *B. adolescentis*, *B. bifidum*, and *A. putredinis*, showed significant negative correlations with TC, TG, and LDL-C (Figures 4B,D). Conversely, the dyslipidemia-enriched species, such as *Prevotella* sp. AM42-24, *Veillonella parvula*, *Bacteroides* sp. CAG:530, *Prevotella* sp. CAG:520, was positively associated with TC, TG, and LDL-C. Specifically, *Faecalibacterium* and *F. prausnitzii* were significantly negatively associated with HDL-C but significantly positively associated with TC (*p* < 0.05, Figures 4B,D).

### Functional disparities between two cohorts and their associations with serum lipid profiles

**Figure 5:**
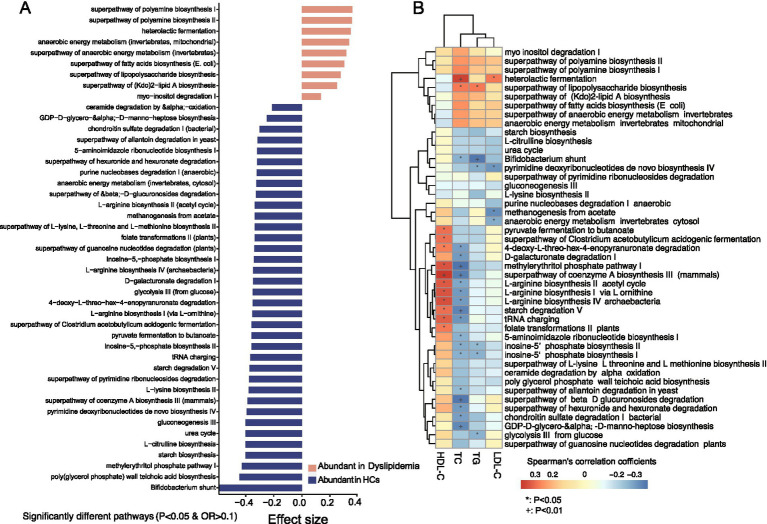
Predicted functional pathways analysis and their association with blood lipid levels. **(A)** There are significantly different predicted functional pathways between the two groups. **(B)** Association analysis between the significantly different functional pathways and blood lipid levels.

HUMAnN 3.0 analysis annotated a total of 411 predicted functional pathways, 45 of which exhibited significant differences between the two groups (Figure 5A and Supplementary Table S1I). In dyslipidemic patients, nine pathways were significantly enriched, including heterolactic fermentation, myo-inositol degradation I, superpathway of (Kdo)_2_-lipid A, LPS, and fatty acids biosynthesis, anaerobic energy metabolism, and superpathway of polyamine biosynthesis I/II (Figure 5A). Conversely, 36 predicted pathways were more abundant in HCs, encompassing Bifidobacterium shunt, biosynthesis of amino acids (L-lysine, L-arginine, L-citrulline, L-threonine, and L-methionine); carbohydrate metabolism pathways (starch biosynthesis and degradation V, pyruvate fermentation to butanoate, D-galacturonate degradation I, glycolysis III (from glucose), and the superpathway of *β*-D-glucuronoside degradation); the superpathway of *Clostridium acetobutylicum* acidogenic fermentation; urea cycle; poly(glycerol phosphate) wall teichoic acid biosynthesis; gluconeogenesis III; pyrimidine and purine metabolism; folate transformations; methanogenesis from acetate; anaerobic energy metabolism (invertebrates, cytosol), and chondroitin sulfate degradation I (bacterial), as well as GDP-D-glycero-*α*-D-manno-heptose biosynthesis (Figure 5A).

To further clarify the intricate interactions between gut microbiota and their functional roles, we employed HUMAnN 3.0 with the UniProt Reference Clusters (UniRef50) database, identifying a total of 11,126 predicted pathway-species associations. Among these, 487 associations were significantly enriched in dyslipidemic patients, while 694 were more abundant in HCs (Supplementary Table S1J). Overall, 66 species were involved in 176 pathways, with 22 of these species showing significant differences between groups: six were enriched in dyslipidemic patients, and 16 were enriched in HCs. Notably, the primary contributors to the majority of predicted functional pathways included *Parabacteroides distasonis* (65 pathways), *Faecalibacterium prausnitzii* (54 pathways), *Bifidobacterium adolescentis* (45 pathways), *Bacteroides xylanisolvens* (44 pathways), *Bifidobacterium bifidum* (40 pathways), *Bifidobacterium longum* (40 pathways), and *Prevotella* sp. AM42-24 (33 pathways) (Supplementary Figures S2–S8). These species were primarily associated with functional pathways related to nutrient metabolism and energy metabolism.

In the analysis of associations between predicted functional pathways and blood lipids (Supplementary Table S1K), several pathways with reduced abundance in dyslipidemic patients—including pyruvate fermentation to butanoate, the superpathway of *Clostridium acetobutylicum* acidogenic fermentation, D-galacturonate degradation I, 4-deoxy-L-threo-hex-4-enopyranuronate degradation, the methylerythritol phosphate pathway I, the superpathway of coenzyme A biosynthesis III (mammals), L-arginine biosynthesis II (acetyl cycle), L-arginine biosynthesis I (via L-ornithine), L-arginine biosynthesis IV (archaebacteria), and starch degradation V—exhibited negative correlations with TC. Notably, *F. prausnitzii*, *P. distasonis*, *B. adolescentis*, *B. xylanisolvens*, *B. bifidum*, *B. longum*, and *Prevotella* sp. AM42-24 were identified as major contributors to these pathways (Figure 5B).

## Discussion

We employed shotgun metagenomic sequencing to investigate the gut microbial composition and function in high-altitude plateau residents with and without hyperlipidemia. Our results revealed a significant reduction in gut microbial diversity among dyslipidemic patients compared to HCs. Furthermore, individuals with hyperlipidemia exhibited a notable decrease in beneficial species, including *F. prausnitzii*, *B. adolescentis*, *B. longum*, *B. bifidum*, and *B. pseudocatenulatum*, as well as functional pathways related to carbohydrate metabolism and amino acid biosynthesis. Conversely, *Prevotella* sp. AM42-24 and pathways involved in the biosynthesis of LPS, fatty acids, and lipid A were significantly enriched in the dyslipidemic group. Importantly, these microbial and functional alterations were found to be associated with blood lipid levels.

In our study, Firmicutes and Bacteroidetes stood out as the two most abundant bacterial phyla in plateau-dwelling individuals. The phylum Firmicutes excels at degrading dietary fiber and fats, while the phylum Bacteroides specializes in breaking down animal proteins (19). Notably, studies have reported that Bacteroides species in Tibetans exhibit higher expression levels of fatty acid oxidase genes, which enables them to efficiently metabolize the saturated fats abundant in yak meat—a staple in the traditional Tibetan diet—thereby mitigating intestinal barrier damage that would otherwise result from fat accumulation (20). Specifically, the Firmicutes/Bacteroidetes (F/B) ratio was determined to be 1.31 in HCs and 1.61 in patients with dyslipidemia, suggesting a significant correlation between hyperlipidemia and the F/B ratio. Notably, an elevated F/B ratio is commonly associated with obesity, while a decreased ratio is linked to inflammatory bowel disease (21). Furthermore, research has documented an increased F/B ratio in both humans with hyperlipidemia (22) and experimental animals with the condition (23). Additionally, our study observed a significant reduction in the abundance of Actinobacteria among plateau residents with dyslipidemia. This phylum is well recognized for its exceptional metabolic versatility and its role as a major source of clinically used antibiotics (24). The enrichment of the gram-negative gut bacterial phylum Bacteroidetes (relative abundance: dyslipidemic patients vs. HCs = 40:35), coupled with reduced levels of the gram-positive phyla Firmicutes (relative abundance: dyslipidemic patients vs. HCs = 52:56) and Actinobacteria in dyslipidemic patients, suggests that the gut microbiota in these individuals may produce increased amounts of lipopolysaccharide (LPS). This elevation in LPS could potentially disrupt the intestinal barrier.

In plateau-dwelling patients with dyslipidemia, the abundance of opportunistic bacteria was found to be increased. *Prevotella* was identified as the most prevalent genus among plateau residents, with no significant differences in its overall abundance between dyslipidemic patients and HCs. *Prevotella* plays a crucial role in the metabolism of carbohydrates, lipids, and amino acids in the host, and its relative abundance was significantly associated with the growth performance of livestock (25). Oral intake of *Prevotella* can ameliorate the lipid metabolism of laying hens and this beneficial effect of *Prevotella* is consistent across different species (26). However, our study revealed a significant elevation in two specific strains—*Prevotella* sp. AM42-24 and *Prevotella* sp. CAG:520—in plateau dyslipidemic patients, both of which are involved in numerous microbial functional pathways. The genus *Prevotella* comprises over 50 characterized species that inhabit diverse natural environments, with most species associated with humans (27). While generally regarded as harmless commensals, these strains possess various virulence factors, including adhesins, hemolysins, secretion systems, exopolysaccharides, LPS, proteases, quorum-sensing molecules, and antibiotic resistance mechanisms. These factors enable them to evolve into well-adapted pathogens capable of successfully infecting and proliferating within host tissues (28). Additionally, other opportunistic pathogens were found to be increased in plateau dyslipidemic patients, including *Streptococcus oralis* (29), *S. mitis* (30), *Veillonella* spp., and *Clostridium*. *Streptococcus* is a risk factor for lipoprotein metabolism disorders, and *Streptococcus anginosus* could induce TG metabolism disorders by impairing the utilization of dietary triglycerides.^1^ The genus *Veillonella* includes 16 characterized species, eight of which are commonly found in the human oral cavity (31). *Veillonella* spp. are involved in certain human infections, and their antibiotic susceptibility and biofilm-forming ability play important roles in oral biofilm ecology (32). For instance, *V. tobetsuensis* is an anaerobic, gram-negative coccus isolated from human tongue biofilms (33), while *V. parvula* promotes intestinal inflammation by activating macrophages via the LPS-TLR4 pathway (34). Recent studies have indicated that gut *Veillonella* promotes human homeostasis by producing beneficial metabolites, specifically short-chain fatty acids (SCFAs), by lactate fermentation (35). *Clostridium* can break down the complex polysaccharides in highland barley to generate short-chain fatty acids (SCFA), such as butyric acid, which can promote the repair of the intestinal barrier. Notably, these hyperlipidemia-enriched bacterial species showed a significant positive correlation with the levels of TC, TG, and HDL-C.

In plateau-dwelling patients with dyslipidemia, the abundance of beneficial gut bacterial species was significantly reduced. Notably, *Faecalibacterium* (the second most prevalent genus) and its species *F. prausnitzii* (the second most abundant species) were both markedly decreased in these patients. As a next-generation probiotic, *F. prausnitzii* is known to produce butyrate and promote the growth of short-chain fatty acid (SCFA)-producing bacteria (36). It is also recognized as an anti-inflammatory commensal bacterium, identified through the gut microbiota analysis of Crohn’s disease patients (37). Moreover, *F. prausnitzii* has been reported to improve lipid metabolism disorder and insulin resistance in type 2 diabetic mice (38), and *F. prausnitzii* treatment improves hepatic health and reduces adipose tissue inflammation in high-fat-fed mice (39). Thus, its reduction may potentially contribute to the development of hyperlipidemia in high-altitude residents.

In addition, compared to HCs, dyslipidemic patients showed significantly lower RA of *Bifidobacterium* and *Alistipes*. *Bifidobacterium* and *Roseburia* are butyrate producers that can provide energy for intestinal epithelial cells to promote the repair of tight junctions (40). Our study detected a notable decrease in *B. adolescentis*, *B. longum*, *B. bifidum*, and *Bifidobacterium pseudocatenulatum* among plateau dyslipidemic patients. The genus *Bifidobacterium* is characterized by its early colonization of the human gastrointestinal tract and potential health-promoting effects on the host—properties that have led to its inclusion as an active ingredient in various functional foods (41), and *Bifidobacterium* was reported to participate in lipid metabolism by regulating intestinal flora, producing SCFAs and bile salt hydrolase (BSH) (42). Among these species, *B. adolescentis*, a key member of the human gut microbiota involved in GABA production (43) and a potential modulator of gut–brain axis interactions, exhibited significantly reduced abundance in plateau dyslipidemic patients. *B. bifidum* species, meanwhile, display distinct physiological and genetic traits, including epithelial adhesion and metabolism of host-derived glycans (44). Additionally, *B. longum*, a recognized probiotic with reported protective effects against inflammatory bowel disease (45), was also significantly diminished in plateau hyperlipidemia patients. Interestingly, our study observed a significant reduction in *Alistipes putredinis*, *A. indistinctus*, and *A. shahii* in plateau dyslipidemic patients. The genus *Alistipes* comprises 13 species, predominantly found in the human gut microbiome (46, 47). While certain studies suggest that specific *Alistipes* species may act as pathogens in conditions like colorectal cancer and depressive symptoms, conflicting evidence indicates their potential protective roles against liver fibrosis, colitis, cancer immunotherapy resistance, and cardiovascular disease (46). This duality implies that reduced *Alistipes* abundance could contribute to the development of liver-related disorders.

Beyond the aforementioned species belonging to *Faecalibacterium*, *Bifidobacterium*, and *Alistipes*, we also observed other bacterial shifts in dyslipidemic patients. Among these, the saccharolytic bacterium *Parabacteroides distasonis* exhibited a significant reduction in abundance in plateau-dwelling dyslipidemic patients and was involved in the biosynthesis of ribonucleotides and amino acids. *P. distasonis* is capable of metabolizing carbohydrates such as mannose and raffinose to produce SCFAs, including acetate, propionate, and succinate, to serve as energy sources for intestinal epithelial cells and shape microbial interactions (63). Notably, it can potentially improve insulin resistance by activating intestinal GPR109a (48). Additionally, *P. distasonis* modulates host metabolism and alleviates obesity and metabolic dysfunctions through the production of succinate and secondary bile acids (49). Another bacterium, *Bacteroides xylanisolvens*, showed a significant decrease in abundance among plateau individuals with hyperlipidemia. *B. xylanisolvens* produced certain beneficial metabolites, including *γ*-aminobutyric acid, SCFAs, and tryptophan metabolites, to enhance host health (50). Studies have demonstrated that oral administration of live *B. xylanisolvens* reduces hepatic steatosis and enhances folate-mediated signaling pathways in mice, while knockout of the folate biosynthetic gene *folP* in *B. xylanisolvens* abolishes its folate production and the associated beneficial effects. These findings confirm the therapeutic potential of *B. xylanisolvens* in alleviating non-alcoholic hepatic steatosis and provide evidence for the beneficial role of the gut *Bacteroides*-folate-liver axis (51).

Significant functional differences in gut microbiota were observed between the two groups. Notably, in plateau dyslipidemic patients, pathways such as polyamine biosynthesis, heterolactic fermentation, anaerobic energy metabolism (invertebrate mitochondrial), the superpathway of fatty acid/LPS/(Kdo)₂-lipid A biosynthesis, and myo-inositol degradation were significantly enriched. Polyamines play a critical role in energy metabolism, with particular relevance to the regulation of lipid metabolism, making them a key focus for research into obesity and related disorders (52). Polyamine biosynthesis was primarily driven by microbial species, including *Escherichia coli*, *Klebsiella pneumoniae*, *Klebsiella* var*iicola*, *Mitsuokella jalaludinii*, *Mitsuokella multacida*, and *Raoultella planticola*, all of which showed no significant differences between the two groups. Heterolactic metabolism primarily produces lactate, acetate, or ethanol as metabolites (53). (Kdo)₂-lipid A (3-deoxy-d-manno-octulosonic acid-lipid A) is an essential component of lipopolysaccharide (LPS) in most Gram-negative bacteria and represents the minimal structural unit required for bacterial viability. It acts as the active moiety of LPS, triggering robust host immune responses through interactions with the Toll-like receptor 4 (TLR4)-myeloid differentiation protein 2 complex (54). Both heterolactic fermentation and (Kdo)₂-lipid A biosynthesis were participated by *E. coli* and *K. pneumoniae*. Fatty acid biosynthesis initiation was participated by dyslipidemia-abundant species, including *Veillonella atypica*, *Veillonella infantium*, *V. parvula*, *F. prausnitzii*, *Lactobacillus delbrueckii*, *Streptococcus vestibularis*, and other species, including *E. coli*. Interestingly, the superpathway of lipopolysaccharide biosynthesis is significantly increased in dyslipidemic patients and is participated by *E. coli*. Lipopolysaccharides (LPSs) are bacterial surface glycolipids, produced by Gram-negative bacteria, which are known to determine acute inflammatory reactions and trigger chronic inflammation (55).

Conversely, other functional pathways—including gluconeogenesis, glycolysis, starch degradation, pyruvate fermentation to butanoate, and the biosynthesis of L-arginine, L-lysine, L-citrulline, L-threonine, and L-methionine—were downregulated in plateau dyslipidemic patients. These pathways were primarily associated with HC-enriched species, including *Alistipes shahii*, *A. putredinis*, *A. indistinctus*, *Alistipes Finegoldii*, *Anaerostipes hadrus*, *Coprococcus catus*, *F. prausnitzii*, *Bifidobacterium bifidum*, *B. adolescentis*, *B. angulatum*, *B. catenulatum*, *Parabacteroides distasonis*, *P. merdae*, *Catenibacterium mitsuokai*, *Dorea longicatena*, *Gemmiger formicilis*, *Ruthenibacterium lactatiformans*, *Blautia wexlerae*, *Eubacterium hallii*, *Blautia obeum*, *Bacteroides thetaiotaomicron*, *B. fragilis*, and *Lactococcus lactis*. Interestingly, these pathways showed a significant positive correlation with HDL-C and a negative correlation with TC. Accumulating experimental and clinical evidence suggests that L-arginine supplementation may aid in managing metabolic disturbances in obesity, regulating arterial blood pressure, or alleviating symptoms of type 2 diabetes, though the underlying mechanisms remain incompletely understood and are currently under investigation (56). Metabolites of the lysine pathway have also been linked to the risk of type 2 diabetes and cardiovascular disease (57). L-citrulline, a neutral *α*-amino acid abundant in watermelon, is synthesized by mitochondrial enzymes and serves as a substrate for L-arginine recycling (58). Supplementation with L-citrulline has shown potential as an intervention to lower blood pressure (59). In hyperlipidemic rats fed a high-fat diet, L-threonine levels in adipose tissues were elevated following the consumption of hawthorn ethanol extracts (60). Additionally, L-threonine has been found to extend health span in *Caenorhabditis elegans* by accelerating the ferritin-dependent inhibition of ferroptosis (61). Regarding methionine, supplementation has been associated with oxidative stress and proteasome dysfunction, leading to hepatotoxicity and hepatic inflammation in rats. However, the relationships between methionine, oxidative stress, proteasome function, and liver injury require further clarification (62).

Our study identified unique gut microbial community structures and functional pathways in dyslipidemic patients residing in high-altitude pastoral areas, with these microbial features being associated with either host lipid metabolism or intestinal barrier impairment. While this study yielded valuable and insightful findings, several limitations warrant consideration in future research endeavors. First, considering the high prevalence of dyslipidemia in high-altitude regions, the sample size employed in the current study is relatively small, which may restrict the statistical power and generalizability of the results. Second, the study samples were solely collected from two pastoral areas within the Gannan Tibetan Autonomous Prefecture, leading to potential geographic bias and limiting the extrapolation of conclusions to other Tibetan pastoral populations. In subsequent studies, we plan to expand the sample cohort by collecting additional specimens from multiple Tibetan pastoral areas. This expansion will help validate the current findings, enhance the robustness of our conclusions and ultimately provide a more solid theoretical foundation for the application of gut microbiota in the diagnosis and treatment of dyslipidemia among Tibetan pastoralists.

## Conclusion

In conclusion, we identified significant changes in gut microbiota composition and function, and their associations with blood lipid levels, in plateau-dwelling dyslipidemic patients. Beneficial species (e.g., *B. adolescentis*, *F. prausnitzii*, *B. longum*, and *B. bifidum*) were reduced, while opportunistic pathogens (*Prevotella* sp. AM42-24, *V. parvula*, *V. tobetsuensis*, *S. oralis*, and *S. mitis*) increased significantly. Dyslipidemic patients showed enriched functional pathways for biosynthesis of LPS, fatty acids, polyamines, and (Kdo)₂-lipid A, whereas healthy controls had enriched carbohydrate metabolism and biosynthesis of L-arginine, L-citrulline, L-lysine, L-threonine, and L-methionine. These findings not only address the research gap in lipid metabolism and gut microbiota under the conditions of “special populations + special environments” but also provide a reference for metabolic studies on populations residing in extreme environments worldwide and lay a scientific foundation for the precise prevention and control of hyperlipidemia in pastoral areas.

## Data Availability

The shotgun metagenomic sequencing data that support the findings of this study are available at: https://www.ncbi.nlm.nih.gov, accession number PRJNA1067529.

## Footnotes

^1^ https://www.researchsquare.com/article/rs-7055704/v1
