# Supplementary material for: Structural and functional characterization of gut microbiota in dyslipidemic patients from high-altitude Tibetan pastoral areas
Source: Front Nutr. 2025 Oct 8;12:1676238. doi: 10.3389/fnut.2025.1676238 (PMC12542731; doi:10.3389/fnut.2025.1676238)
Supplement: Supplementary file 1 [file Data_Sheet_1.PDF]

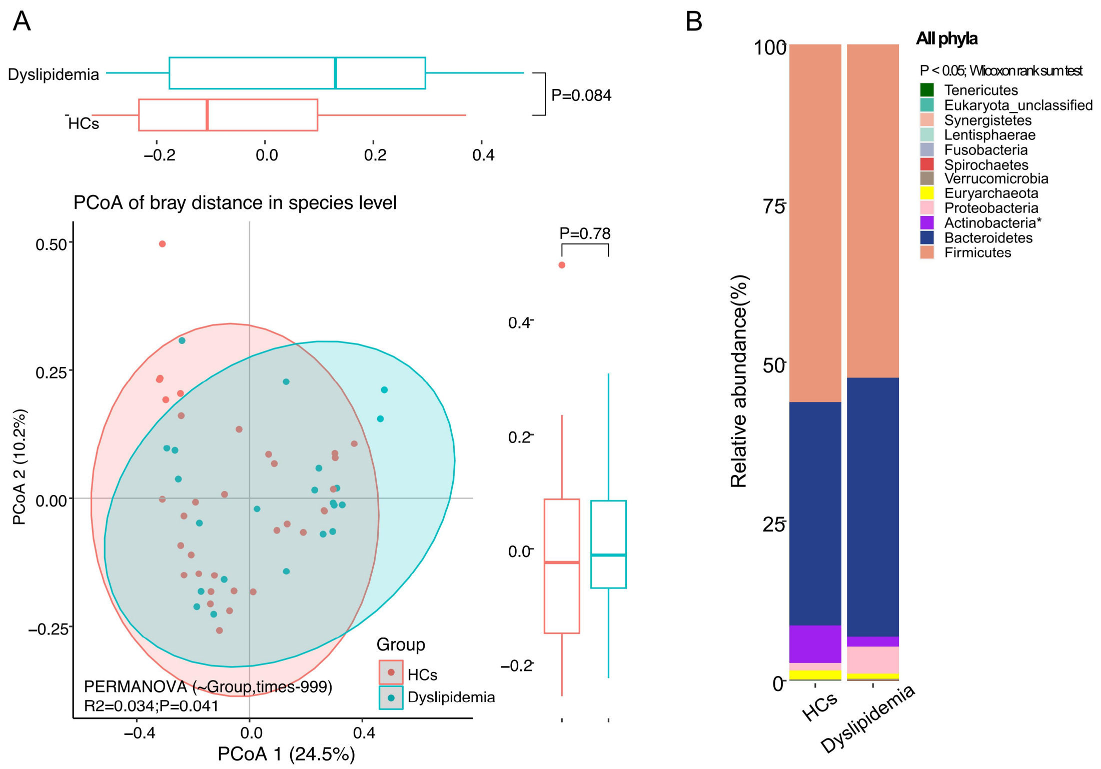

Figure S1. The blood lipids in hyperlipidemic patients and healthy controls.

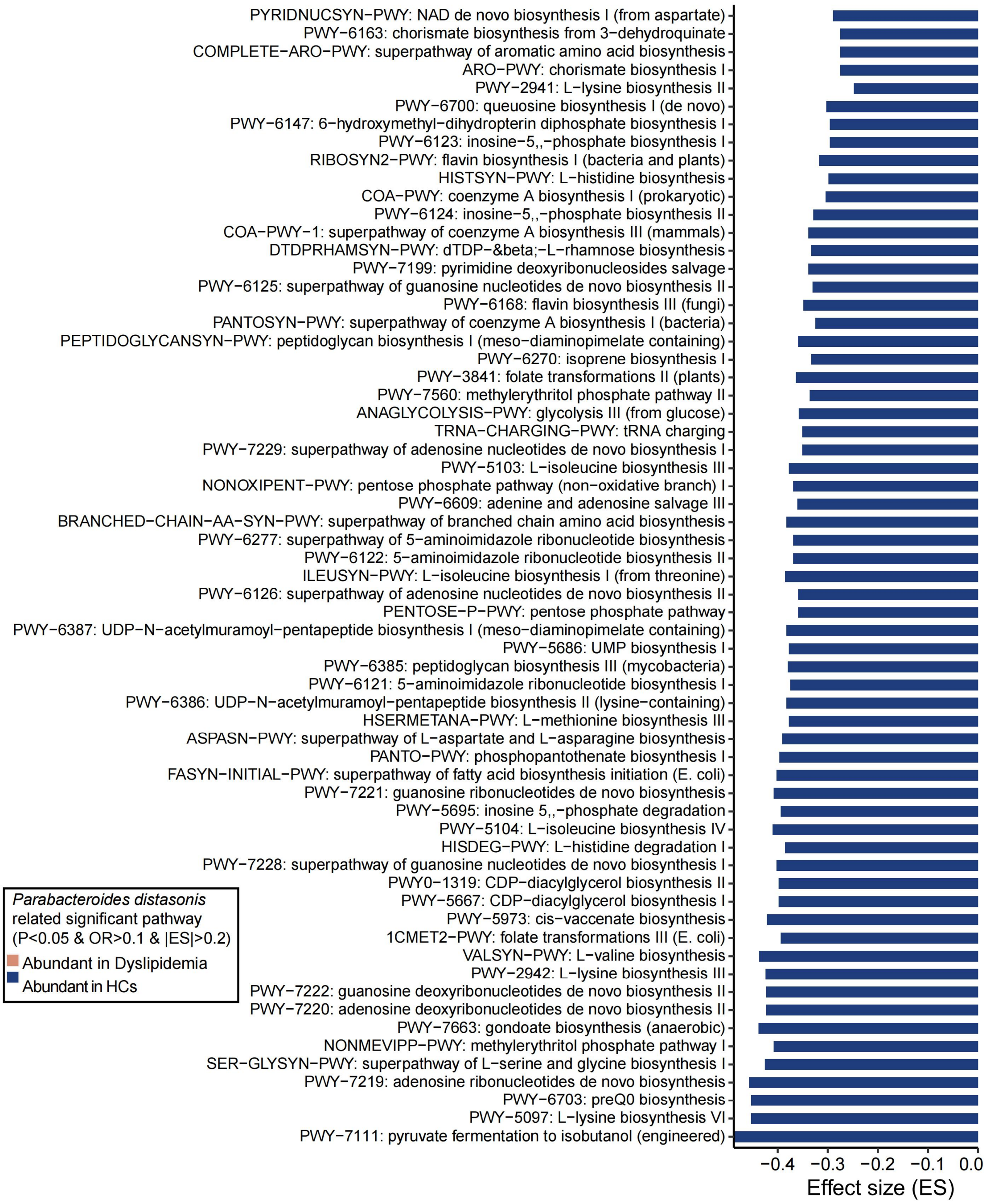

Figure S2. Predicted functional pathways involved for *Parabacteroides distasonis*.

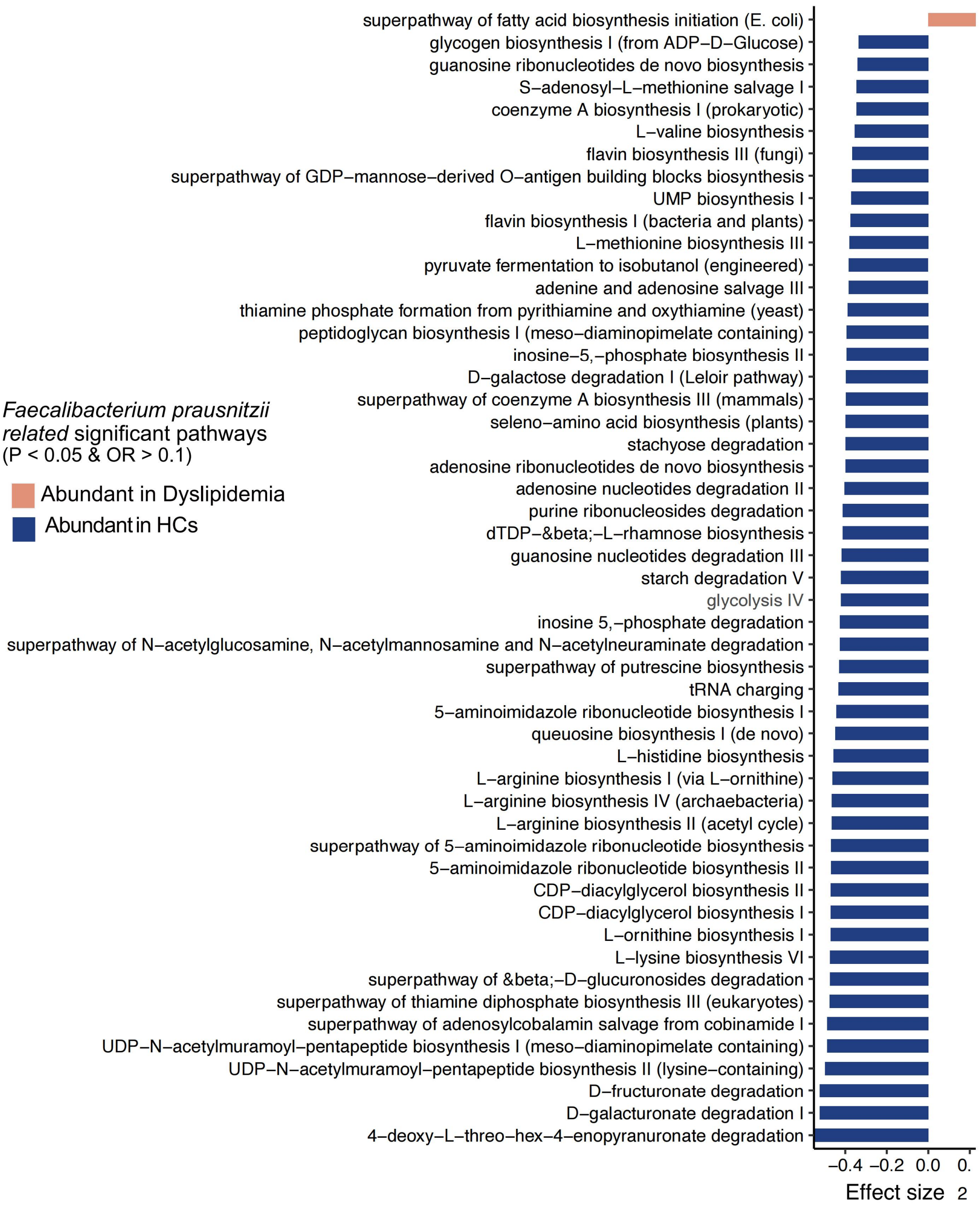

Figure S3. Predicted functional pathways involved for *Faecalibacterium prausnitzii*.

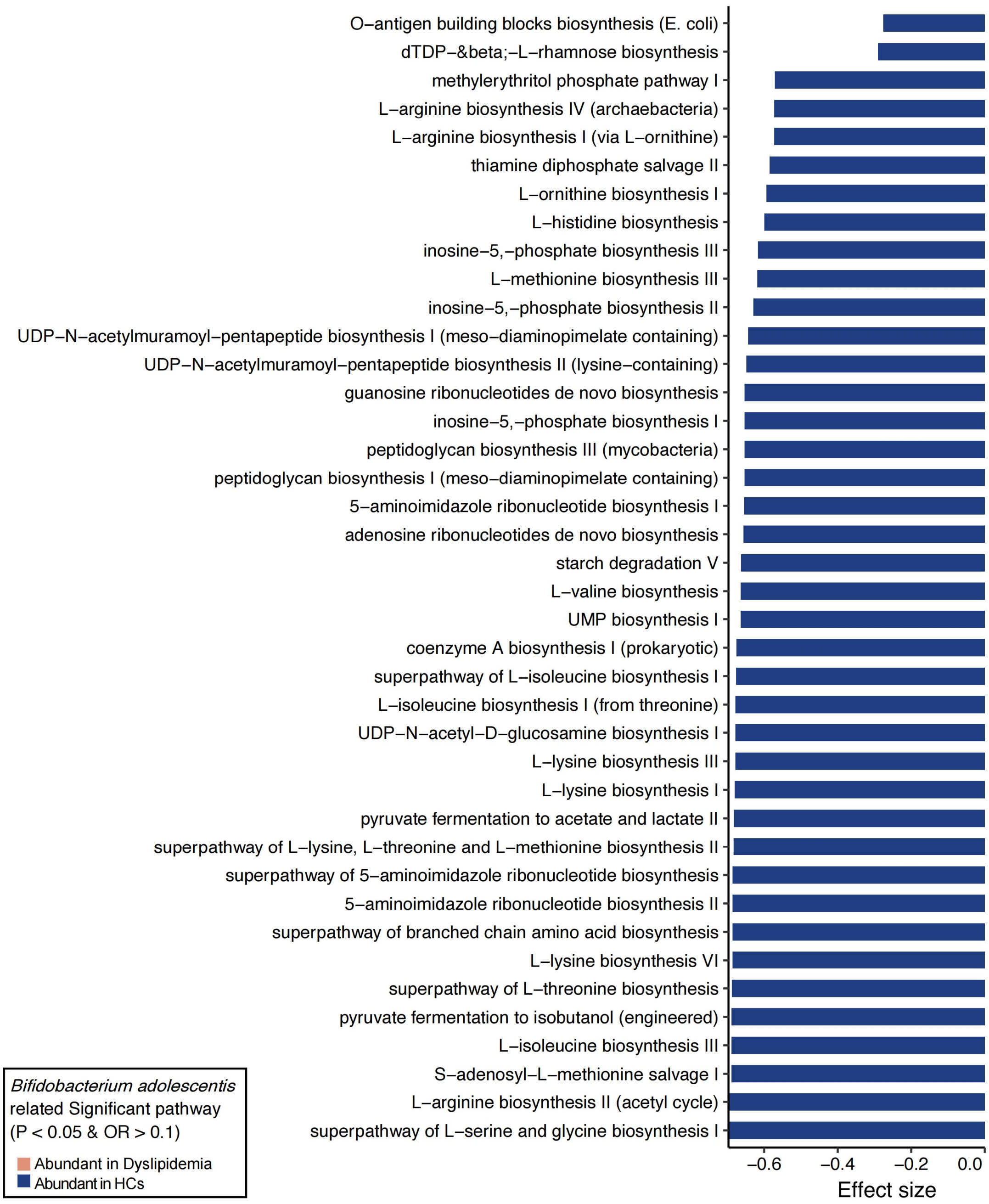

Figure S4. Predicted functional pathways involved for *Bifidobacterium adolescentis*.

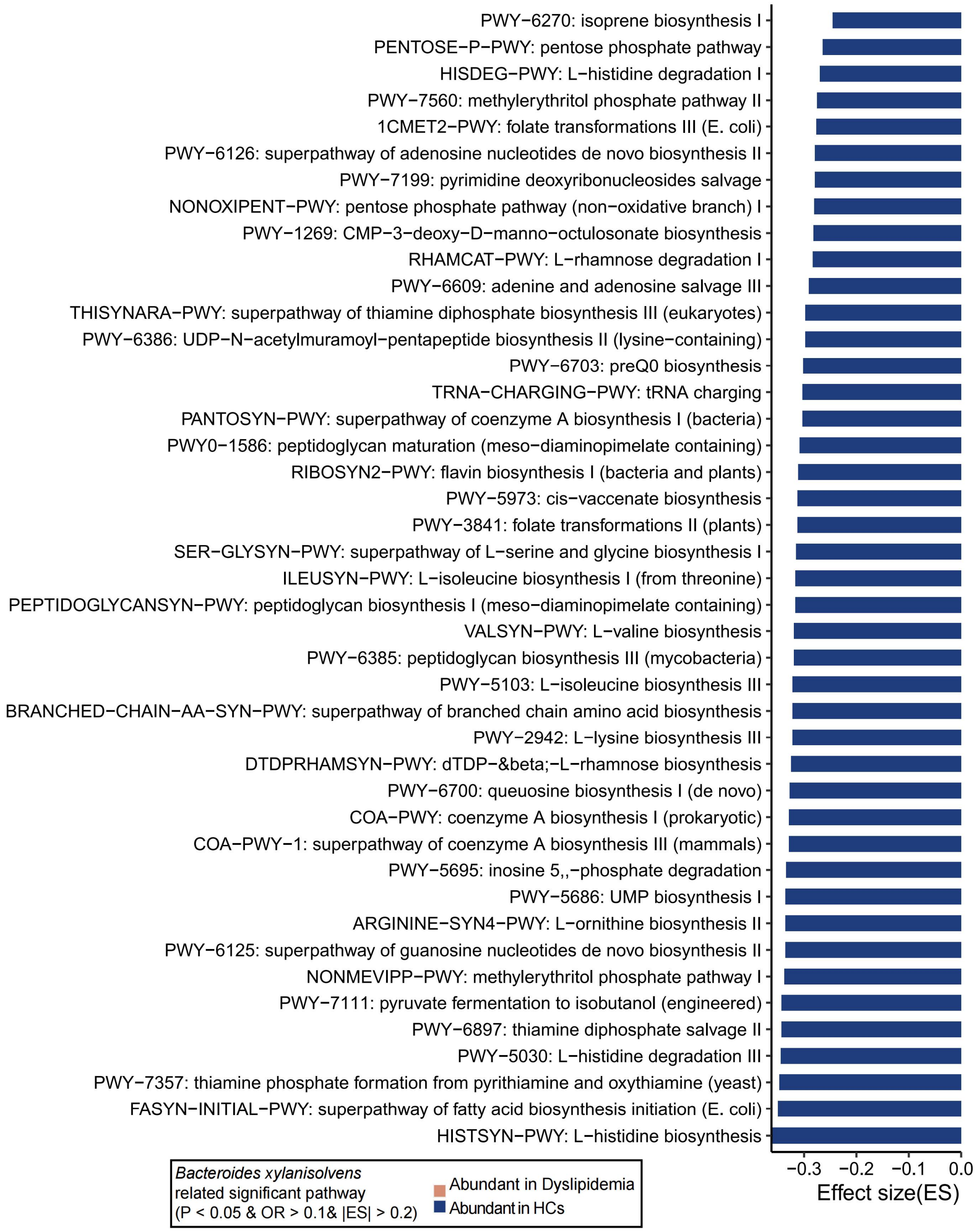

Figure S5. Predicted functional pathways involved for *Bacteroides xylanisolvens*.

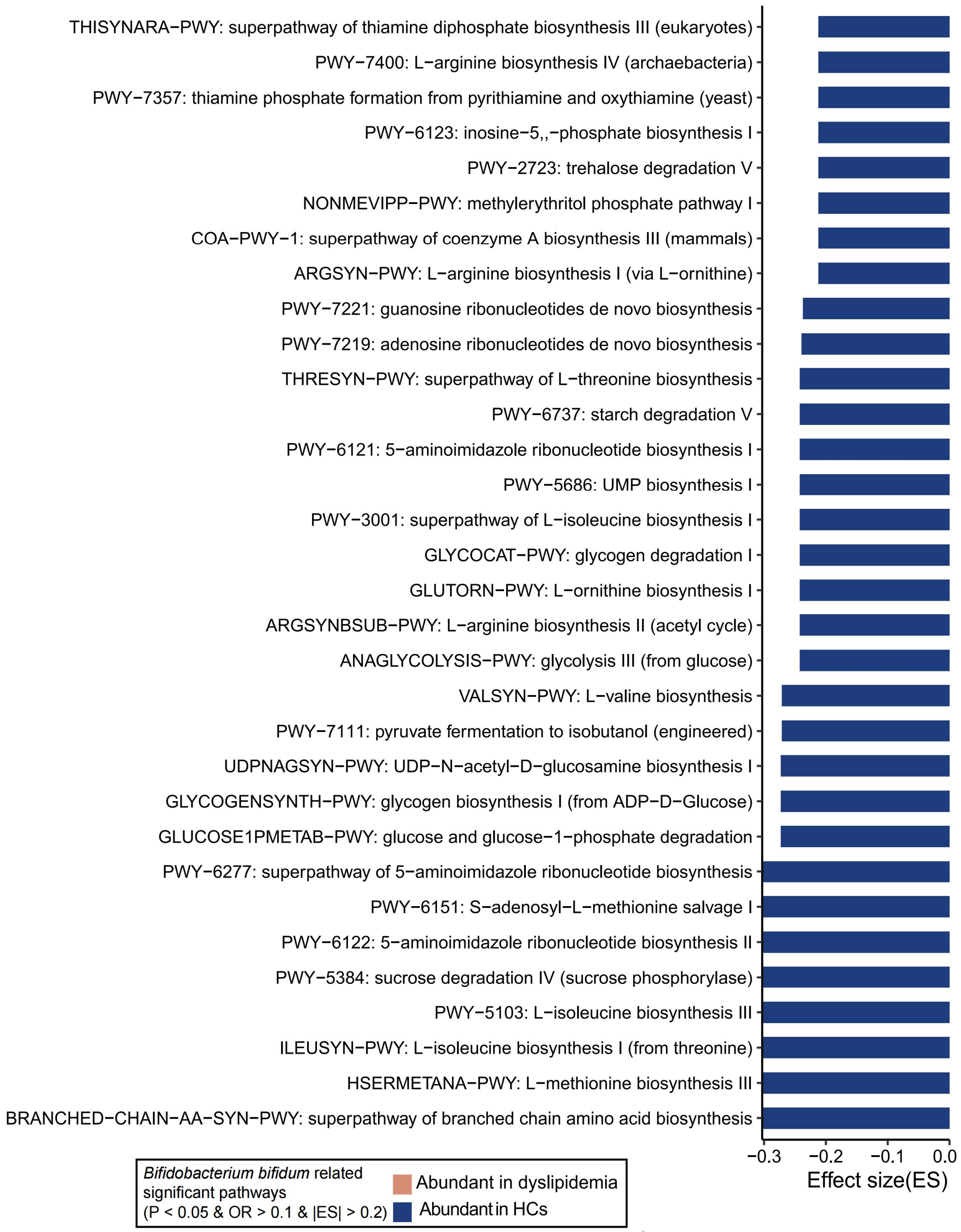

Figure S6. Predicted functional pathways involved for *Bifidobacterium bifidum*.

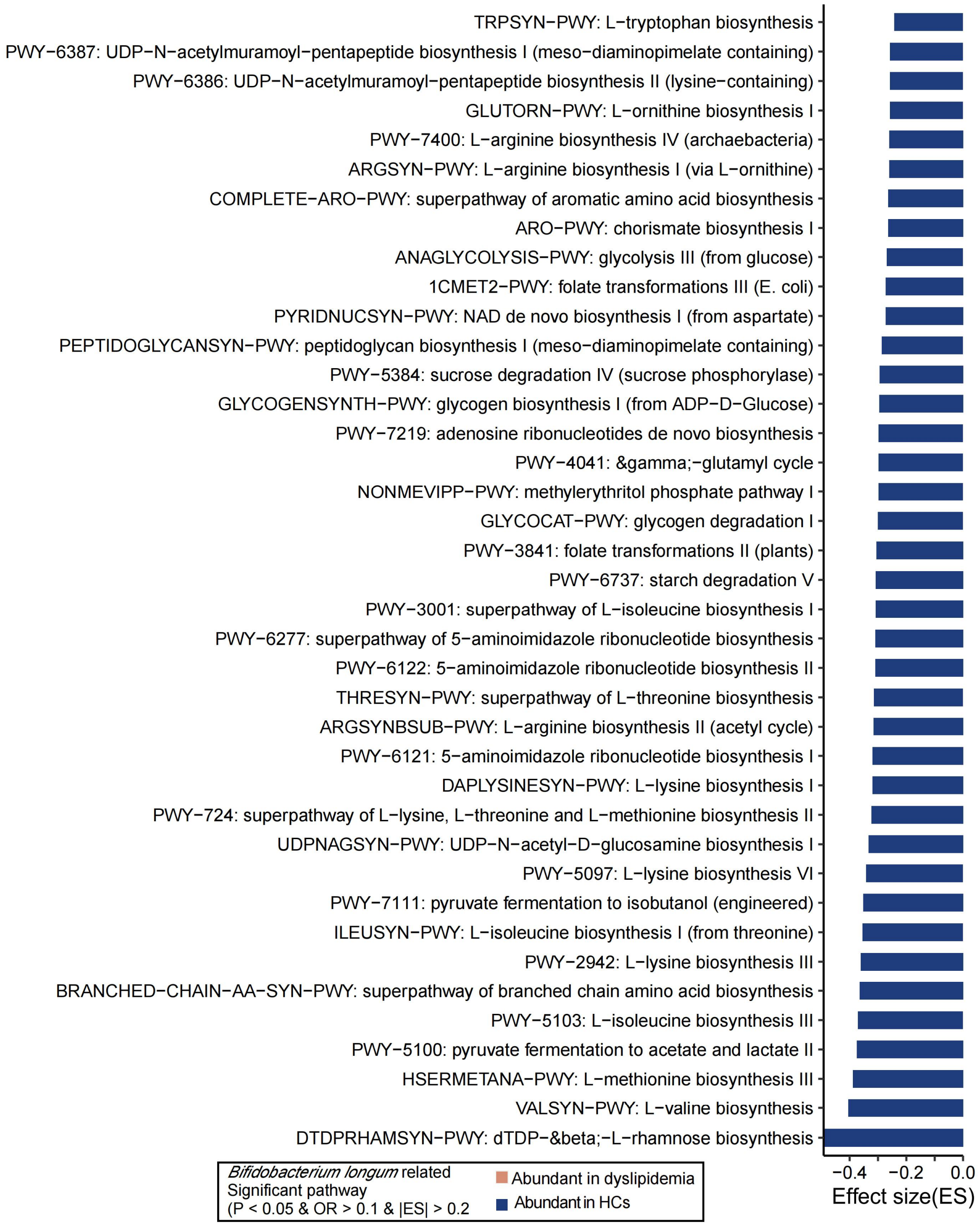

Figure S7. Predicted functional pathways involved for *Bifidobacterium longum*.

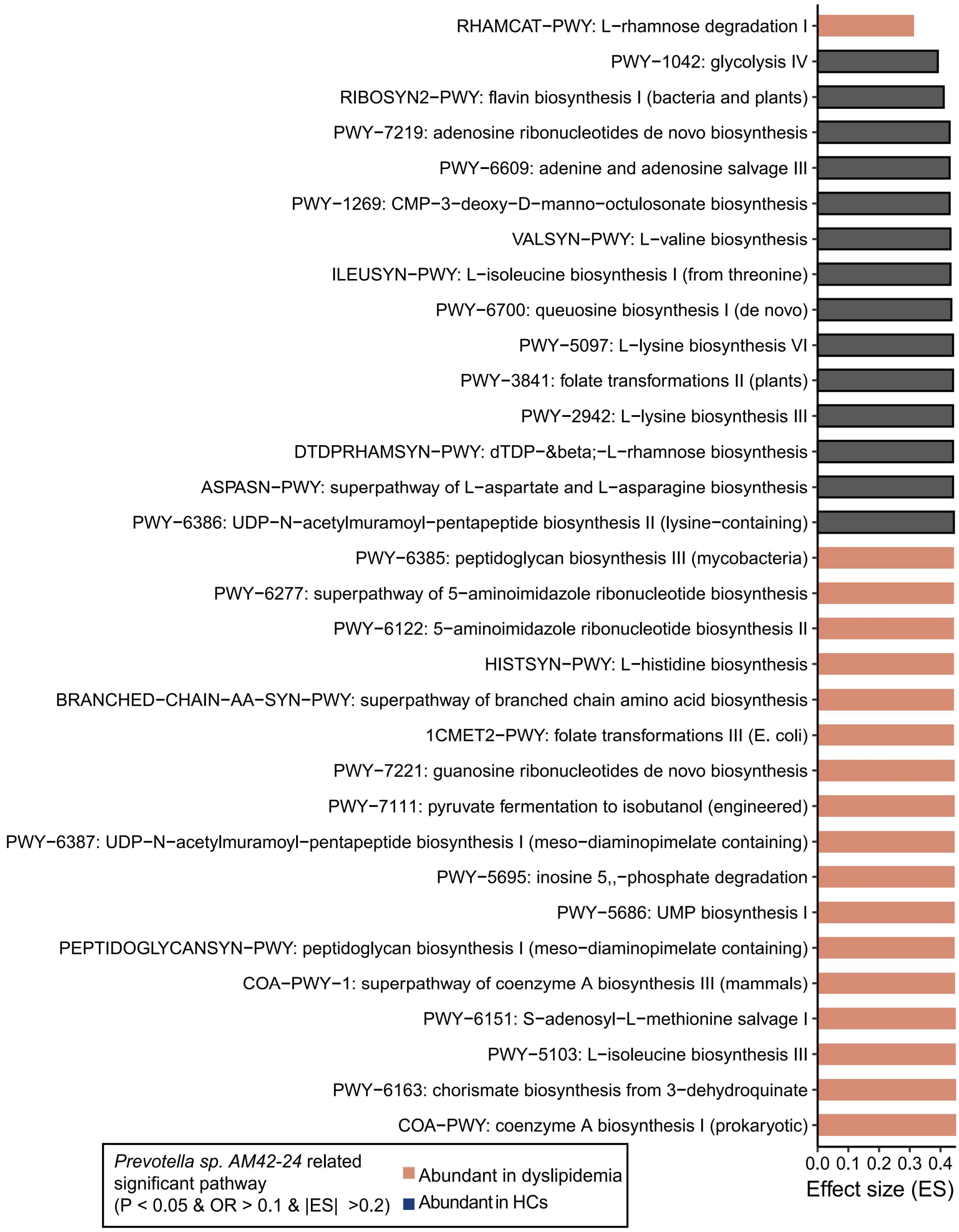

Figure S8. Predicted functional pathways involved for *Prevotella* sp. AM42:24.
